# Clinical implications of genomic profiles in metastatic breast cancer with a focus on TP53 and PIK3CA, the most frequently mutated genes

**DOI:** 10.18632/oncotarget.15881

**Published:** 2017-03-03

**Authors:** Ji-Yeon Kim, Eunjin Lee, Kyunghee Park, Woong-Yang Park, Hae Hyun Jung, Jin Seok Ahn, Young-Hyuck Im, Yeon Hee Park

**Affiliations:** ^1^ Division of Hematology-Oncology, Department of Medicine, Samsung Medical Center, Sungkyunkwan University School of Medicine, Seoul, 06351, Korea; ^2^ Samsung Genome Institute, Samsung Medical Center, Sungkyunkwan University School of Medicine, Seoul, 06351, Korea; ^3^ Biomedical Research Institute, Samsung Medical Center, Sungkyunkwan University School of Medicine, Seoul, 06351, Korea; ^4^ Samsung Advanced Institute for Health Sciences and Technology, Sungkyunkwan University School of Medicine, Seoul, 06351, Korea

**Keywords:** metastatic, breast cancer, TP53, PIK3CA

## Abstract

Breast cancer (BC) has been genetically profiled through large-scale genome analyses. However, the role and clinical implications of genetic alterations in metastatic BC (MBC) have not been evaluated. Therefore, we conducted whole-exome sequencing (WES) and RNA-Seq of 37 MBC samples and targeted deep sequencing of another 29 MBCs. We evaluated somatic mutations from WES and targeted sequencing and assessed gene expression and performed pathway analysis from RNA-Seq. In this analysis, *PIK3CA* was the most commonly mutated gene in estrogen receptor (ER)-positive BC, while in ER-negative BC, *TP53* was the most commonly mutated gene (*p* = 0.018 and *p* < 0.001, respectively). *TP53* stopgain/loss and frameshift mutation was related to low expression of *TP53* in contrast nonsynonymous mutation was related to high expression. The impact of *TP53* mutation on clinical outcome varied with regard to ER status. In ER-positive BCs, wild type *TP53* had a better prognosis than mutated *TP53* (median overall survival (OS) (wild type vs. mutated): 88.5 ± 54.4 vs. 32.6 ± 10.7 (months), *p* = 0.002). In contrast, mutated *TP53* had a protective effect in ER-negative BCs (median OS: 0.10 vs. 32.6 ± 8.2, *p* = 0.026). However, *PIK3CA* mutation did not affect patient survival. In gene expression analysis, *CALM1*, a potential regulator of *AKT*, was highly expressed in *PIK3CA*-mutated BCs. In conclusion, mutation of *TP53* was associated with expression status and affect clinical outcome according to ER status in MBC. Although mutation of *PIK3CA* was not related to survival in this study, mutation of *PIK3CA* altered the expression of other genes and pathways including *CALM1* and may be a potential predictive marker of PI3K inhibitor effectiveness.

## INTRODUCTION

Breast cancer (BC) is the most common malignancy in women worldwide [1]. Recent advances in therapeutic strategies have improved BC-specific mortality and morbidity, but still only one-quarter of metastatic BC patients survive until 5 years after BC diagnosis [2].

In the era of next-generation sequencing (NGS), numerous genetic alterations causing BC have been discovered. The Cancer Genome Atlas (TCGA) consortium performed comprehensive genetic analysis of BCs [3]. They showed that *TP53, PIK3CA*, and *GATA3* were the genes most commonly mutated, and that genetic alterations differed according to BC subtype (luminal A, B, basal-like, or HER2-enriched). The International Cancer Genome Consortium (ICGC) reported that 93 protein coding cancer genes carried driver mutations [4]. Similar to TCGA results, genetic alterations differed according to BC subtype; *TP53, PTEN*, and *RB1* were the genes most frequently mutated in estrogen receptor (ER)-negative BC, whereas *PIK3CA, CCND1*, and *GATA3* were rarely mutated in ER-negative BC.

Many clinical trials based on these mutated genes have been proposed and are on-going [5]. *PIK3CA* is considered a targetable potential driver of BC. *PI3K*α and *PIK3δ* inhibitors and *AKT* inhibitors are being used to treat ER-positive BC patients harboring *PIK3CA* mutations in clinical trials [6–8]. Recently, everolimus, an mTOR inhibitor, was approved for postmenopausal ER-positive metastatic BCs [9, 10]. An additional biomarker study showed that BC patients with mutated *PIK3CA* derived clinical benefit from everolimus; however, BC patients with wild-type *PIK3CA* also responded to everolimus [11].

Here, we identified gene alterations in MBC using whole-exome and whole-transcriptome sequencing. We evaluated mutation profiles and expression patterns and analyzed the relationship between genetic alterations and expression of specific genes and pathways. Because we performed our large-scale genetic studies using BC surgical specimens, our findings, in addition to describing genetic alterations in advanced BC, could help establish treatment strategies for refractory BC. We conclude by proposing an optimal treatment plan for MBC BCs.

## RESULTS

### Samples and clinical data

We enrolled 54 patients with metastatic BC. Of these 54 patients, RNA sequencing was performed in 37. RNA-Seq was not performed for 17 samples due to RNA extraction failure. The characteristics of the 37 patients are described in Table 1. The median age of enrolled patients was 45.1 years, and 35.1% patients had TNBC. Fourteen of 37 patients (37.8%) had basal-like subtype BC. Five patients were tested for the BRCA1/2 mutation, and a germline BRCA1 and/or BRCA2 mutation was detected in three patients. Visceral metastasis was found in 15 patients, eight patients had brain metastasis, and the others had liver metastasis. All specimens were from biopsy from metastatic BC not archival tissue. Most common biopsy site was breast main mass (32.4%). Patients with metastatic BC received more than three palliative treatments on average. Thirty-six of 37 patients had received anthracycline-containing cytotoxic chemotherapy, and 31 patients were treated with taxane chemotherapy. All ER-positive BCs were treated with tamoxifen or a non-steroidal aromatase inhibitor. Anti-HER2 treatment was administered in all patients with HER2-positive BCs.

**Table 1:** Clinicopathological characteristics of metastatic breast cancer (*N* = 37)

	*N* = 37 (%)
Age (median)	45.1 ± 11.0
Range	26.5–75.7
< 40 years old	15 (40.5)
≥ 40 years old	22 (59.5)
Histology	
Invasive ductal carcinoma	34 (91.9)
Other	3 (8.1)
Subtype	
ER+HER2−	12 (32.4)
ER+HER2+	5 (13.5)
ER-HER2−	13 (35.1)
ER-HER2+	7 (18.9)
Intrinsic subtype	
Luminal A	7 (18.9)
Luminal B	6 (16.2)
Basal-like	14 (37.8)
Normal-like	2 (5.4)
HER2-enriched	8 (21.6)
BRCA1/2	
Wild-type	2 (5.4)
Mutated	3 (8.1)
Not tested	32 (86.5)
Cancer status	
Recurrent	27 (73.0)
Initially metastatic	10 (27.0)
Visceral metastasis	
Yes	15 (40.5)
Liver metastasis	7 (18.9)
Brain metastasis	8 (21.6)
No	22 (59.5)
Biopsy site	
Breast	12 (32.4)
Lymph node	7 (18.9)
Pleura	7 (18.9)
Liver	3 (8.1)
Lung	2 (5.4)
Other	6 (16.2)
Chemotherapy agents (average 3.24)	
1	8 (21.6)
2	11 (29.7)
3	4 (10.8)
≥ 4	14 (37.8)
Chemotherapeutic regimen	
Anthracycline	36 (97.3)
Taxane	31 (83.8)
Both anthracycline and taxane	27 (73.0)
Hormone therapy (*N* = 17)	
Yes	17 (100.0)
No	0 (0.0)
HER2-targeted therapy (*N* = 12)	
Yes	12 (100.0)
No	0 (0.0)

The time elapsed between diagnosis with metastatic breast cancer and RNA-Seq differed according to breast cancer subtype (Table 2). For ER-HER2+ BC, mean time to RNA-Seq was 29.3 months (range 5.5–69.7 months), whereas in ER-HER2- BC, the corresponding time was 4.3 months (range 0.0–36.7 months).

**Table 2:** Previous chemotherapy and time to biopsy according to subtype

Subtype	No. of previous chemotherapy agents	Time to biopsy after metastasis
ER+HER2−	3.5 (range 1–6)	13.6 months (range 0.1–126.0)
ER+HER2+	4.4 (range 1–11)	18.8 months (range 2.4–33.2)
ER-HER2−	2.5 (range 1–6)	4.3 months (range 0.0–36.7)
ER-HER2+	3.4 (range 1–9)	29.3 months (range 5.5–69.7)

### Significantly mutated genes and mRNA expression in metastatic breast cancer

Overall, 34 tumor samples from 37 patients were subjected to whole-exome sequencing, resulting in identification of 3,278 somatic mutations comprising 3,069 point mutations (single nucleotide variants; SNVs) and 209 insertion/deletions. Among the point mutations, 44 were silent mutations, 2,830 were non-synonymous mutations, 184 were stop-gain, and 11 were stop-loss mutations. In addition, 136 frameshift deletions and 73 insertions were detected.

**Figure 1:**
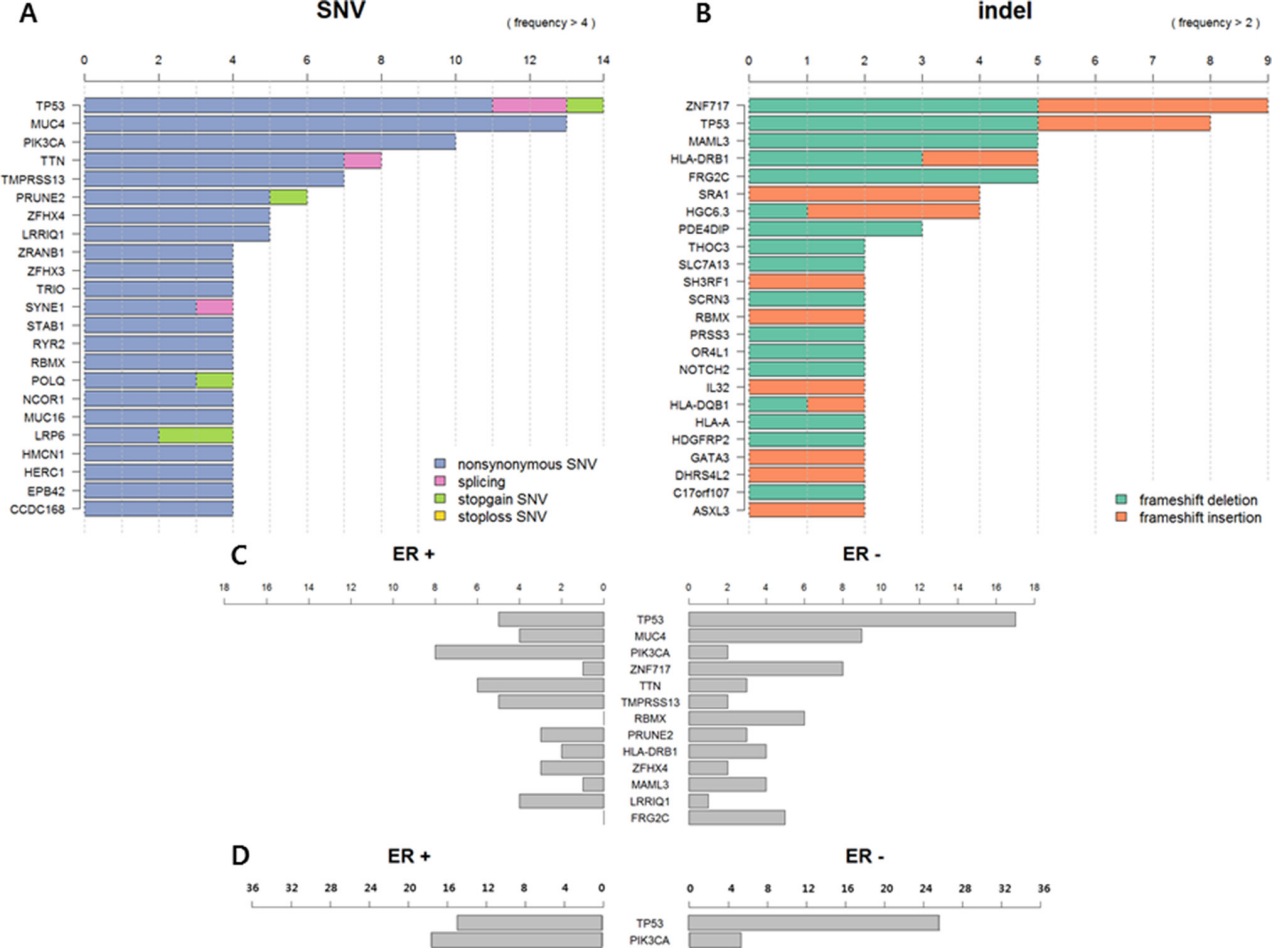
(**A**) Frequency of single nucleotide variants (SNVs) in metastatic breast cancer (BC) (*N* = 34). (**B**) Frequency of frame shift insertion/deletions in metastatic BC (*N* = 34). (**C**) Somatic mutation profile according to ER status in metastatic BC (*N* = 34). (**D**) TP53 and PIK3CA mutation profile according to ER status in metastatic BC (*N* = 63).

*TP53* was the most frequently mutated gene in metastatic BC (64.7%, 14 SNVs, and 8 frameshift insertions and deletions (indels)), followed by *MUC4* (38.2%) and *PIK3CA* (29.4%). Frameshift mutations were most commonly observed in *ZNF717* (26.5%) (Figure 1A and 1B).

Somatic mutations differed according to ER status. *TP53* was more frequently mutated in ER-negative BCs than ER-positive BCs (*p* = 0.126) in contrast to *PIK3CA* (*p* = 0.019). *TTN*, *TMPRESS13*, and *LRRIQ1* were also more frequently mutated in ER-positive BCs, and *ZNF717, RBNX*, and *FRG2C* were more frequently mutated in ER-negative BCs, but there were no significant differences between the two groups (Figure 1C).

We additionally performed targeted deep sequencing of 29 metastatic BC samples to detect *TP53* and *PIK3CA* mutations. Then, we reanalyzed 63 sequences. Of these 63 BCs, 36 (57.1%) were ER-positive (Supplementary Table 3). In this analysis, *TP53* mutation was more frequently detected in ER-negative BC (ER-positive vs. ER-negative: 41.7% vs. 92.6% *p* < 0.001) and *PIK3CA* mutation was more common in ER-positive BC (ER-positive vs. ER-negative: 47.2% vs. 18.5%, *p* = 0.018) (Figure 1D).

Gene expression patterns also differed according to ER status. Among 22,072 genes, *ERBB4, GATA3, FOXA1*, and another 434 genes were more highly expressed in ER-positive BC than ER-negative BC (false discovery rate (FDR) *p* < 0.05 respectively). In contrast, *KRT16, S100A2, RASAL1*, and another 282 genes were more highly up-regulated in ER-negative BC than ER-positive BC (Supplementary Figure 2 and Supplementary Table 1).

### The relationship between somatic mutations and gene expression

Because DNA is ultimately transcribed to messenger RNA followed by protein translation, we analyzed the association between somatic mutation and gene expression using Fisher's exact test and the log-rank test. We focused on the two most commonly mutated genes, *TP53* and *PIK3CA*.

**Figure 2:**
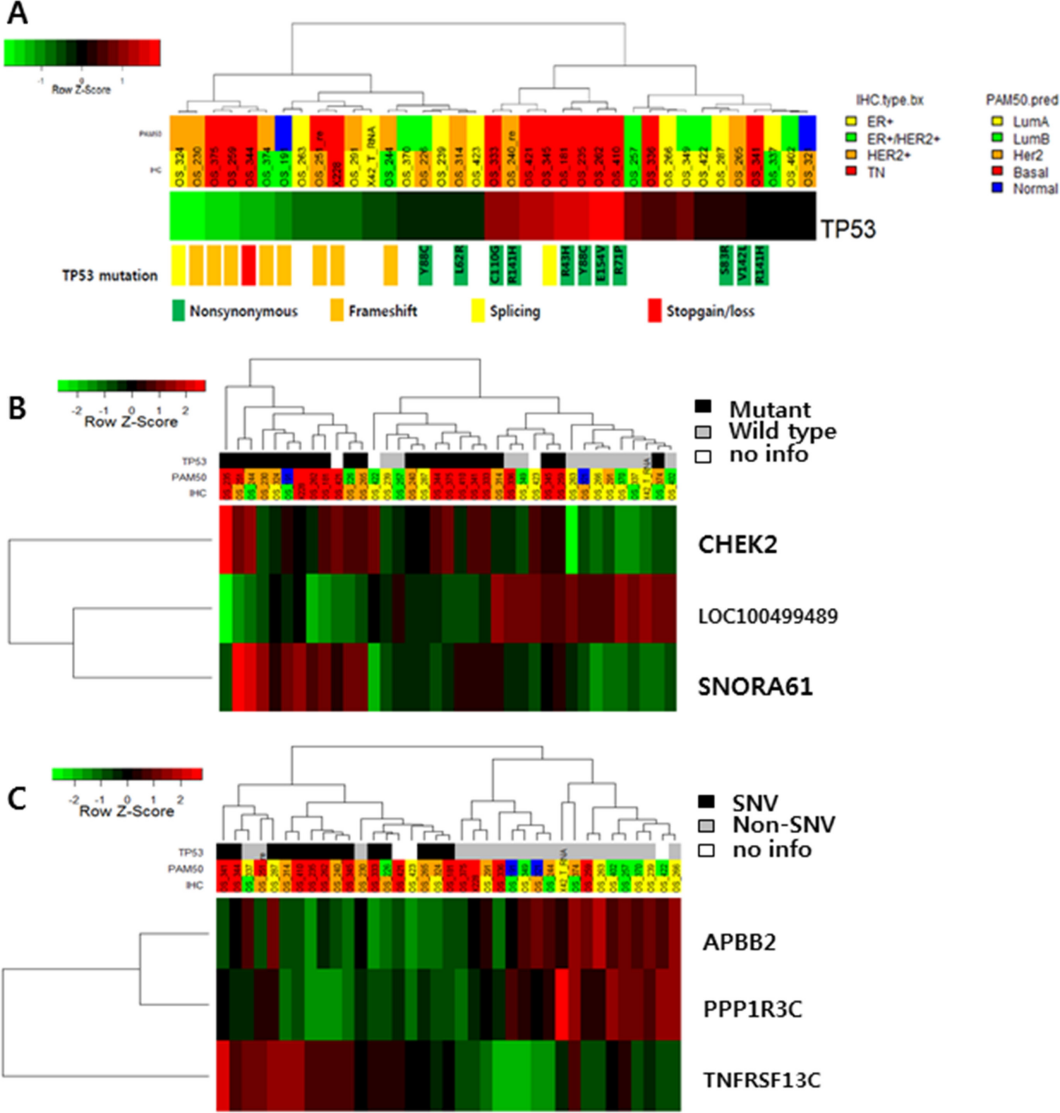
(**A**) *TP53* expression according to *TP53* mutation status. (**B**) Gene expression according to *TP53* mutation status (TOP3 genes: CHEK2, SNORA61 and LOC100499489). (**C**) APBB2, PPP1R, and TNFRSF13C expression according to *TP53* single nucleotide variants.

Mutation of *TP53* affected the level of gene expression (Figure 2A). Frameshift indels and stop-gain mutations of *TP53* decreased gene expression compared to nonsynonymous mutations. In addition, high expression of *CHEK2* and *SNORA61* and low expression of *LOC100499489* were detected in *TP53*-mutated BC (Figure 2B and Supplementary Figure 3). SNVs of *TP53* (only nonsynonymous mutations, not frameshift indels or splicing variants) were associated with low expression of *APBB2* and *PPP1R3C* and high expression of *TNFRSF13C*, but there were no statistically significant differences (Figure 2C).

**Figure 3:**
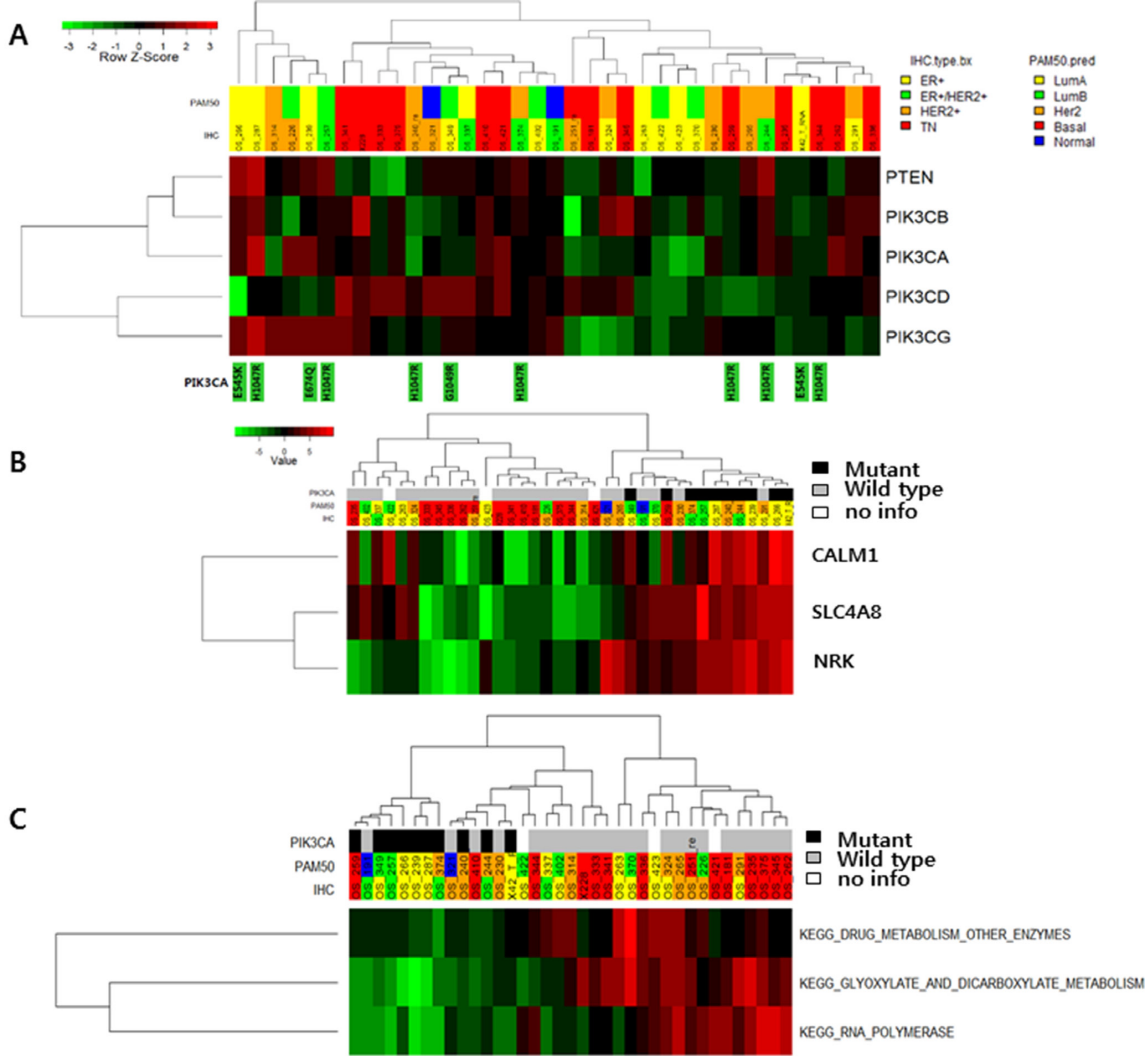
(**A**) Expression of *PIK3CA* pathway-associated genes according to *PIK3CA* mutation status. (**B**) Gene expression according to *PIK3CA* mutation status (TOP3 genes: CHEK2, SNORA61 and LOC100499489). (**C**) Pathway gene expression according to *PIK3CA* mutation status.

*PIK3CA* mutations were all nonsynonymous mutations. Nine of 10 mutation occurred at known hotspots: E545, H1047, and G1049. There was no relationship between mutation of *PIK3CA* and gene expression. We also analyzed *PIK3CB*, *PIK3CD*, *PIK3CG*, and *PTEN*, but did not find a correlation between genetic mutation of these genes and gene expression (Figure 3A). In one BC case with *PIK3CA* mutation, high expression of *CALM1, SLC4A8*, and *NRK* was observed (Figure 3B and Supplementary Figure 4). In pathway analysis, low scores for glyoxylate and dicarboxylate metabolism, drug metabolism, and RNA polymerase were associated with mutation of *PIK3CA* (Figure 3C and Supplementary Table 2).

### The relationship between genetic alterations and clinical outcomes

For analyzing the effect of genetic alterations to clinical outcome, we divided metastatic BC into two subtypes, ER-positive and ER-negative. According to subtypes, we analyzed the relationship between genetic alterations and overall survival.

**Figure 4:**
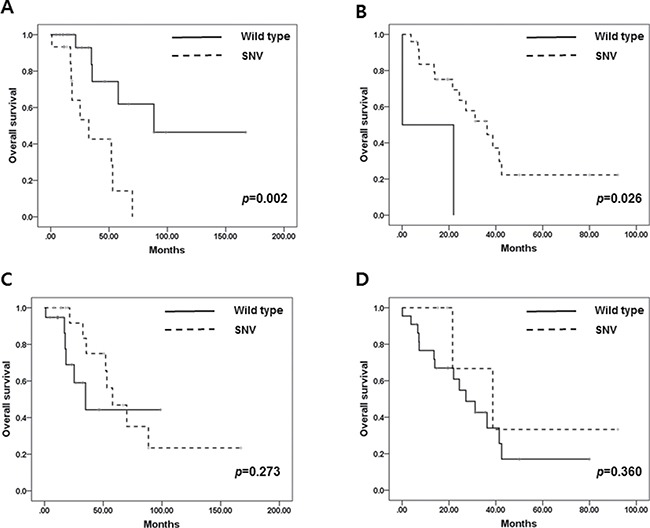
(**A**) Overall survival in ER-positive BC according to *TP53* mutation. (**B**) Overall survival in ER-negative BC according to *TP53* mutation. (**C**) Overall survival in ER-positive BC according to *PIK3CA* mutation. (**D**) Overall survival in ER-negative BC according to *PIK3CA* mutation.

*TP53* mutation was related to shorter OS in ER-positive BC in contrast to ER- negative BC. In ER-positive BC, the median OS of *TP53*-mutated BC was 32.6 months compared to 88.5 months in wild type *TP53* (median OS (wild type vs. mutated): 88.5 ± 54.4 vs. 32.6 ± 10.7 (months), *p* = 0.002). In contrast, *TP53* mutation in ER-negative BC had longer OS compared to wild type *TP53* (median OS (wild type vs. mutated): 0.1 vs. 32.6 ± 8.2 (months), *p* = 0.026) (Figure 4A and 4B). However, there were no significant differences in OS between those with SNVs and frameshift indel mutations.

*PIK3CA* mutation did not affect OS in metastatic BC regardless of ER status (median OS (wild-type vs. mutated): 34.9 ± 11.0 vs. 57.9 ± 12.1 (months), *p* = 0.269 in ER+ BC and median OS: 27.2 ± 5.9 vs. 38.7 ± 14.0 (months), *p* = 0.558 in ER- BC) (Figure 4C and 4D).

## DISCUSSION

We explored genome-wide genetic alterations in metastatic BC in this study and confirmed that *TP53* was the most frequently mutated gene in ER-negative metastatic BCs, while *PIK3CA* was the most frequently mutated gene in ER-positive metastatic BC.

*TP53* mutation, the most common mutation in triple-negative breast cancer [3, 4], was also frequently detected in the metastatic setting. SNVs of *TP53* were commonly observed in TNBC and increased *TP53* mRNA expression. In contrast, *TP53* frameshift indels were more frequently detected in HER2-positive BCs and decreased mRNA expression. Additional gene expression and pathway analysis revealed that SNVs and other types of *TP53* mutations were diversely related to the expression patterns of other genes (Figure 2 and Supplementary Figure 3). Accordingly, *TP53* mutation type affects mutational profile. In a previous study, we reported a relationship between *TP53* mutation profile and expression in TNBCs [12]. Although statistical significance was not reached in the current study because of our small sample size, our results suggest that the *TP53* mutation profile might impact all subtypes of BCs, as well as TNBCs.

We observed *CHEK2* overexpression in TP53-mutated BCs. *CHEK2*, an inducer of the TP53 gene in response to DNA damage, acts as a tumor suppressor. TP53 mutation causes p53 dysfunction and DNA repair system malfunction. Therefore, CHEK2 overexpression may induce CHEK2-mediated DNA repair system activation as a compensatory mechanism in TP53-mutated BC. *TNFRSF13C*, also known as B-cell activating factor receptor (*BAFFR*), was especially activated in metastatic BC with *TP53* SNVs. *TNFRSF13C* mutations have been studied in the context of lymphoid malignancy, with higher expression predicting better prognosis. Further studies to clarify the relationship between *TP53* and *TNFRSF13C* are warranted.

SNVs were detected only in four loci in *PIK3CA*: three hotspots and one rare locus. Similar to previous comprehensive genetic studies of BCs, *PIK3CA* mutations were detected in five ER-positive cases, three ER-positive and HER2-positive cases, one HER2-positive case, and one TNBC case. No correlation between mutation and mRNA expression was observed for *PIK3CA* or *PTEN*. Additional analysis showed that calmodulin 1(*CALM1*) was highly expressed in *PIK3CA*-mutated BCs. Calmodulin, a regulator of calcium metabolism, is thought to be a regulator of AKT activity that indicates poor prognosis [13].

There are several current clinical trials targeting *PIK3CA*. BCs with *PIK3CA* mutation appear to have a poorer prognosis than wild-type BCs, and *PI3K* inhibitors appear to have a clinical benefit regardless of *PIK3CA* mutation status [6]. We propose that mutation of *PIK3CA* alters the activity of other genes involved in tumor aggressiveness, and that inhibiting *PIK3CA* modulates these altered pathways. This would explain why treatment with *PIK3CA* inhibitors improves the outcomes of BC patients with *PIK3CA* mutations as well as those patients without *PIK3CA* mutations. Three pathways related to *PIK3CA* might be targets of *PIK3CA* inhibitors; further validation studies are warranted.

We reviewed the TCGA dataset and found 20 metastatic BCs in the entire TCGA breast cancer cohort (*n* = 1046). The most common mutated gene in TCGA cohort was *PIK3CA* followed by *TP53*. Up to 30% of ER-positive BC had *PIK3CA* mutation (271/537, 53.5%) and 16.8% did *TP53* mutation. Of ER-negative BC, *TP53* was the most commonly mutated gene (154/238, 63.0%). *PIK3CA* was detected in 12.6% of ER-negative BC (30/238) (Supplementary Figure 5A). Among metastatic BCs in TCGA cohort, 3 of 4 ER-negative BC had *TP53* mutation. Five of 16 ER-positive BC had *PIK3CA* mutation (31.3%) and 4 had TP53 mutation (25.0%) (Supplementary 5B). Metastatic BCs in our cohorts had a similar mutation profile to the profile observed in the TCGA cohort. Compared with early BC, metastatic BC more frequently had *TP53* mutations in ER-positive BC. In ER-negative BC, the frequency of *TP53* mutation did not vary between early and metastatic BC. *PIK3CA* mutation was detected at similar rates in early and metastatic BC. Considering TCGA cohort consisting of mainly early BCs, our study would give the novel genomic information of metastatic BCs to solve medical unmet need.

In conclusion, mutation of *TP53*, the most frequent genetic alteration in ER-negative BC, affected gene expression levels. Moreover, the type of *TP53* mutation had a differential influence on clinical outcomes according to ER status. In contrast, there was no association between *PIK3CA* mutation and expression, or of other related genes, namely *PIK3CB, PIK3CD, PIK3CG*, and *PTEN*. Mutation of *PIK3CA* might alter calmodulin expression and other genetic pathways. Further functional validation studies are warranted.

## MATERIALS AND METHODS

### Patients

This study involved prospective explorative analysis of patients with metastatic BC at Samsung Medical Center as an establishing genomic platform for precision medicine in the era of NGS. Women diagnosed with stage IV BC or recurrent BC by diagnostic examination and staging work-up (breast magnetic resonance imaging [MRI], chest computed tomography [CT] scan, abdominal CT scan, bone scan, and/or positron emission tomography [PET]-CT scans if indicated) were included.

All patients provided written informed consent, and study approval was obtained from the Institutional Review Board of Samsung Medical Center, Seoul, Korea (IRB No: SMC 2012-08-065).

### Immunohistochemical (IHC) staining

Two experienced pathologists reviewed all specimens to determine IHC staining for ER, progesterone receptor (PgR), and HER2. ER and PgR positivity were defined using Allred scores ranging from 3 to 8 based on IHC using antibodies to ER (Immunotech, Marseille, France) and PgR (Novocastra Laboratories Ltd., Newcastle upon Tyne, UK). HER2 status was evaluated using a specific antibody (Dako, Glostrop, Denmark) and/or silver *in situ* hybridization (SISH). Grades 0 and 1 for HER2, as assessed by IHC, were defined as a negative result, and grade 3 was defined as a positive result. Amplification of HER2 rated as 2+ by IHC was confirmed by SISH. Ki67 IHC analyses were performed independently using semi-quantitative and quantitative methods (Dako). Triple negativity was defined as a lack of expression of ER, PgR, and HER2.

### DNA and RNA extraction

Unstained sections (4 mm) of tumors consisting of over 75% malignant cells were dissected under microscopy by comparison with an H&E-stained slide, and genomic DNA was extracted using a Qiagen DNA FFPE Tissue kit (Qiagen, Hilden, Germany) according to the manufacturer's instructions. After extraction, DNA concentration and 260/280- and 260/230-nm ratios were measured by spectrophotometry (ND1000, NanoDrop Technologies, ThermoFisher Scientific, MA, USA). Each sample was then quantified using a Qubit fluorometer (Life Technologies, Carlsbad, CA, USA). Libraries were prepared for samples with a genomic DNA total yield > 10 ng.

Areas containing representative invasive breast carcinoma were outlined on the slide. Total RNA was then extracted using a High Pure RNA Paraffin kit (Roche Diagnostic, Mannheim, Germany), and the RNA concentration and 260/280- and 260/230-nm ratios were measured using a NanoDrop ND1000 spectrophotometer (NanoDrop Technologies). Samples were concentrated by a SpeedVacTM concentrator (Thermo Scientific™, Waltham, MA, USA). After concentration, these with less than 1 g/L of total were excluded from downstream analysis.

### Whole-exome sequencing (Table 3)

**Table 3:** Summary of whole-exome sequencing (*N* = 34)

	Min.	1st Qu.	Median	Mean	3rd Qu.	Max.
TotalRead	47,430,000	79,760,000	107,300,000	104,700,000	127,500,000	166,900,000
TotalRead_T	47,430,000	110,100,000	119,300,000	122,300,000	132,900,000	166,900,000
TotalRead_N	50,970,000	71,080,000	80,250,000	87,060,000	97,350,000	146,900,000
Coverage	47.41	92.34	126.4	124.3	150.9	231.1
Coverage_T	47.41	128.8	141.7	143.5	163.7	231.1
Coverage_N	48.83	87.85	93.66	105.1	110.3	193.8
PCT_TARGET_BASES_50X	0.363	0.7602	0.8365	0.7944	0.87	0.939
PCT_TARGET_BASES_50X_T	0.363	0.8436	0.8655	0.8404	0.8835	0.939
PCT_TARGET_BASES_50X_N	0.42	0.7117	0.7658	0.7484	0.8211	0.8733
PCT_TARGET_BASES_100X	0.06776	0.3724	0.5346	0.4986	0.6341	0.7554
PCT_TARGET_BASES_100X_T	0.07207	0.5511	0.6067	0.5836	0.6679	0.7554
PCT_TARGET_BASES_100X_N	0.06776	0.3427	0.38	0.4136	0.4854	0.7329

Poor quality reads were filtered out and aligned to the human reference genome (hg19) using the Burrows-Wheeler Alignment tool (BWA, version 0.7.5a) [14]. To convert Sequence Alignment and Mapping (SAM) files into Binary Alignment and Mapping (BAM) files, we used SAMtools (version 0.1.19) [15]. Polymerase chain reaction (PCR) duplicates were removed from the BAM files by Picard (version 1.93, http://picard.sourceforge.net/) and SAMtools before variant calling. The Genome Analysis Toolkit (GATK, version 2.4.7) [16] was used to recalibrate base quality and optimize local realignment. Single nucleotide variants (SNVs) and indels were called using MuTect (version 1.1.4) [17] and Varscan2 (version 2.3.5) [18] with default parameter settings. Copy number variations were detected with CONTRA (version 2.0.4) [19]. Variants were annotated using ANNOVAR, with gene, chromosomal information, exonic function (synonymous, nonsynonymous, stop gain, non-frameshift, or frameshift indel), amino acid changes, and allele frequencies extracted from public databases such as the 1000 Genomes Project (2012 February version) and dbSNP (version 132).

Variants located in exonic regions with sufficient coverage (minimum depth of coverage ≥ 8) and variant allele frequency (VAF ≥ 0.1) were chosen for further statistical analyses. Synonymous variants were filtered out. Read alignments were manually investigated using the Integrative Genomic Viewer (http://www.broadinstitute.org/igv/).

Fisher's exact test was used to analyze mutations and polymorphic variants separately to discover variants enriched in patients with a favorable outcome. *P*-values < 0.05 were considered significantly different. R version 3.0.2 (http://www.R-project.org/) and R package (ggplot2) were used for all statistical analyses and generation of heat maps and plots.

### RNA-Seq analysis and normalization (Table 4)

**Table 4:** Summary of RNA-Seq (*N* = 37)

	Min.	1st Qu.	Median	Mean	3rd Qu.	Max.
PF_ALIGNED_BASES	3,426,000,000	4,818,000,000	5,413,000,000	5,377,000,000	5,814,000,000	6,976,000,000
CODING_BASES	1,202,000,000	2,220,000,000	2,624,000,000	2,574,000,000	3,008,000,000	3,350,000,000
PCT_CODING_BASES	0.226	0.4499	0.5045	0.4808	0.5289	0.599
PCT_UTR_BASES	0.2149	0.2881	0.3209	0.3194	0.3483	0.415
PCT_INTRONIC_BASES	0.02611	0.03425	0.04298	0.0453	0.05247	0.08502
PCT_INTERGENIC_BASES	0.0573	0.09025	0.1271	0.1545	0.1617	0.5088
PCT_MRNA_BASES	0.4408	0.7687	0.8373	0.8002	0.8652	0.9136
MEDIAN_CV_COVERAGE	0.494	0.5271	0.5776	0.5939	0.6472	0.753

After trimming poor-quality bases from FASTQ files obtained from whole-transcriptome sequencing, we aligned the reads to the human reference genome hg19 with Tophat (version 2.0.6) [20] and performed reference-guided assembly of transcripts with Cufflinks (version 2.1.1) [21]. Alignment quality was verified with SAMtools (version 0.1.19). Transcript abundance was estimated using a count-based method with htseq-count. Gene counts were used as the input for TMM (Trimmed Mean of M values) normalization by the R package edgeR [22], and normalized counts were transformed to log2-counts per million (logCPM) by applying voom from the R package limma [23] to account for higher variability at low expression levels. Genes with zero read counts across all samples were removed for a more powerful statistical test. Pathway analysis was performed using GSVA [24] (Supplementary Figure 1).

### Targeted deep sequencing of *TP53* and *PIK3CA*

We used CancerScan™ to detect *TP53* and *PIK3CA* mutation. After enriched exome libraries were multiplexed, the libraries were sequenced on a HiSeq 2500 sequencing platform (Illumina). Briefly, a paired-end DNA sequencing library was prepared through gDNA shearing, end-repair, A-tailing, paired-end adapter ligation, and amplification. After hybridization of the library with bait sequences for 27 hours, the captured library was purified and amplified with an index barcode tag, and the library quality and quantity were assessed. Sequencing of the exome library was performed using the 100-bp paired-end mode of the TruSeq Rapid PE ClusterKit and TruSeq Rapid SBS Kit (Illumina).

### Intrinsic subtyping

We performed intrinsic subtyping with log-scaled normalized expression values using the 50-gene Prediction Analysis of Microarray (PAM50) subtype predictor as described by Parker et al. [25]. The PAM50 subtype predictor classified tumors into the following groups: luminal A, luminal B, HER2-enriched, basal-like, and normal-like (Supplementary Figure 1).

### Survival analysis

We evaluated the association between gene expression and overall survival (OS) using the R package RcmdrPlugin.survival. OS was defined as the time elapsed between the date of stage IV breast cancer diagnosis and the date of death. For each gene, patients were grouped based on the normalized expression value of the gene, with the top 50% and the bottom 50% representing high and low expression groups, respectively. Survival curves for the two groups were estimated with the Kaplan-Meier method, and the log-rank test was used to compare overall survival curves between the two groups (*p* < 0.05). After pathway analysis with GSVA, Fisher's exact test was used to identify pathways that were enriched with genes significantly associated with overall survival (*p* < 0.05).

## SUPPLEMENTARY MATERIALS FIGURES AND TABLES




