# Supplementary material for: Clinical implications of genomic profiles in metastatic breast cancer with a focus on TP53 and PIK3CA, the most frequently mutated genes
Source: Oncotarget. 2017 Mar 3;8(17):27997–8007. doi: 10.18632/oncotarget.15881 (PMC5438625; doi:10.18632/oncotarget.15881)
Supplement: Supplementary file 1 [file oncotarget-08-27997-s001.pdf]

Clinical implications of genomic profiles in metastatic breast cancer with a focus on TP53 and PIK3CA, the most frequently mutated genes

Supplementary Materials

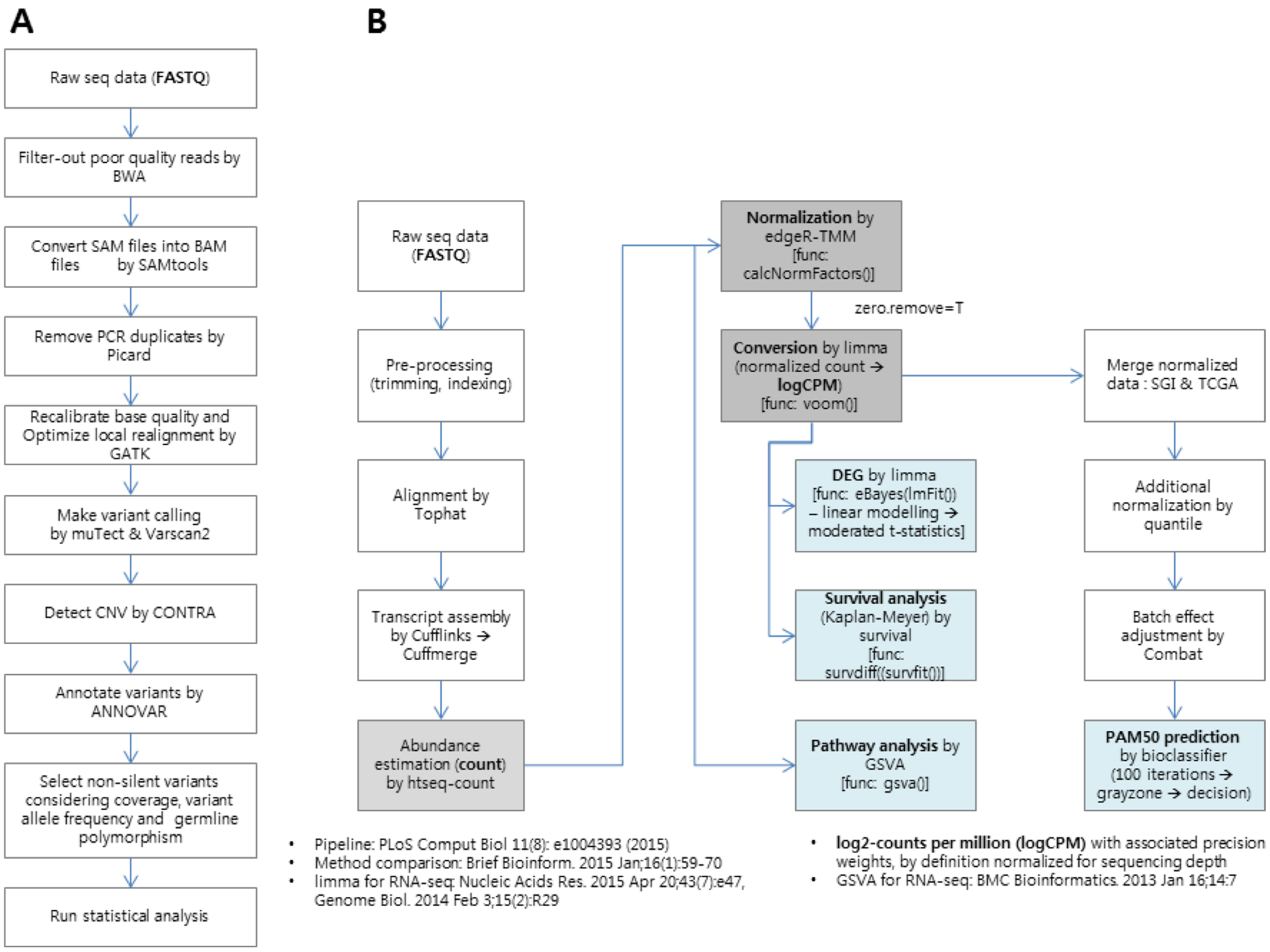

Supplementary Figure 1: Pipeline of next generation sequencing data analysis (**A**) whole exome sequencing (**B**) RNA-Seq.

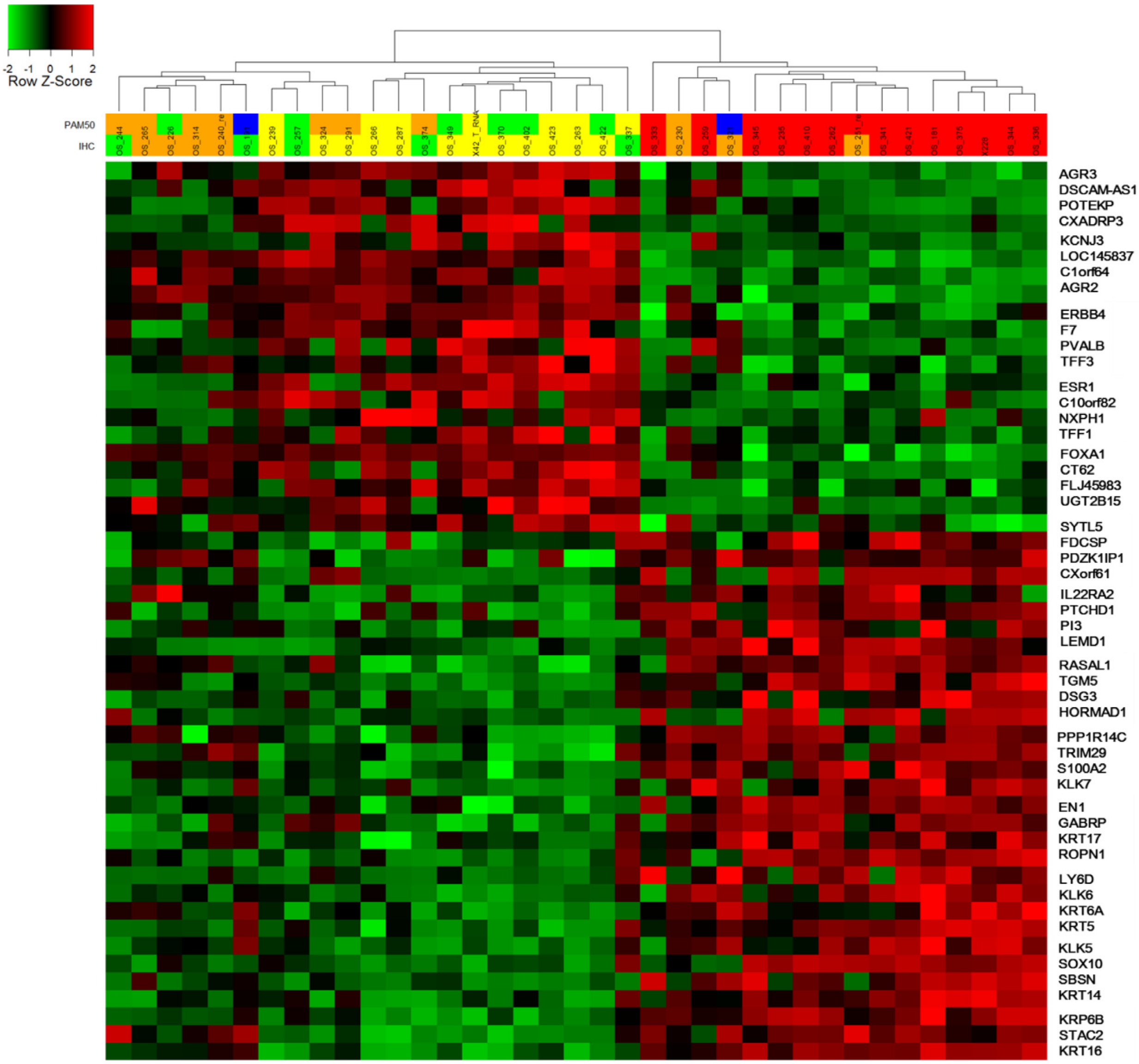

Supplementary Figure 2: RNA expression profile of metastatic breast cancer.

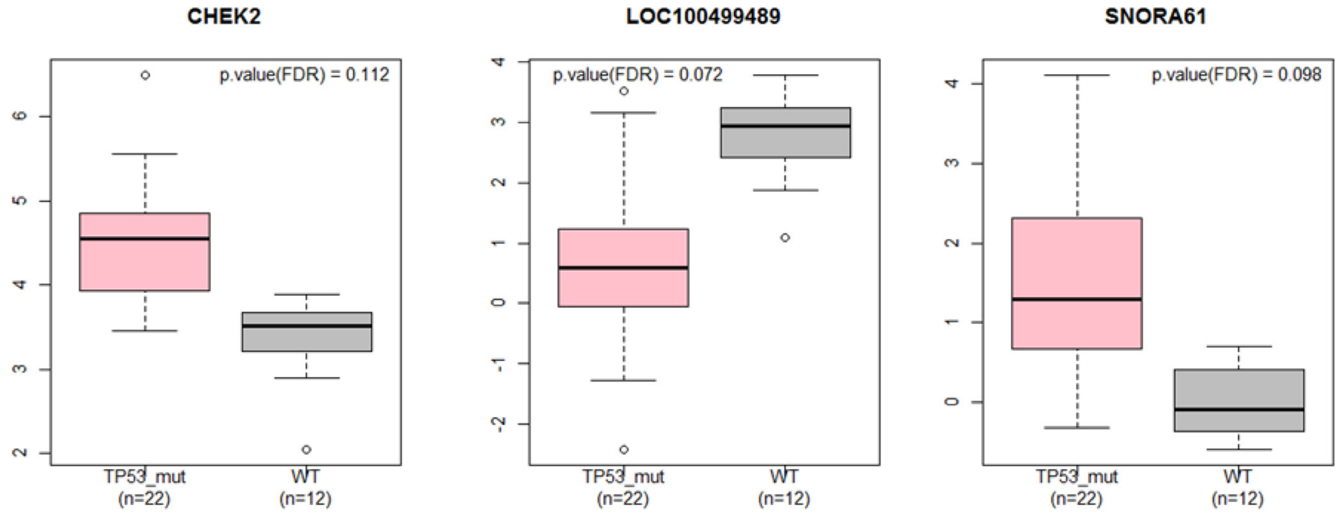

Supplementary Figure 3: The level of gene expression according to *TP53* mutation.

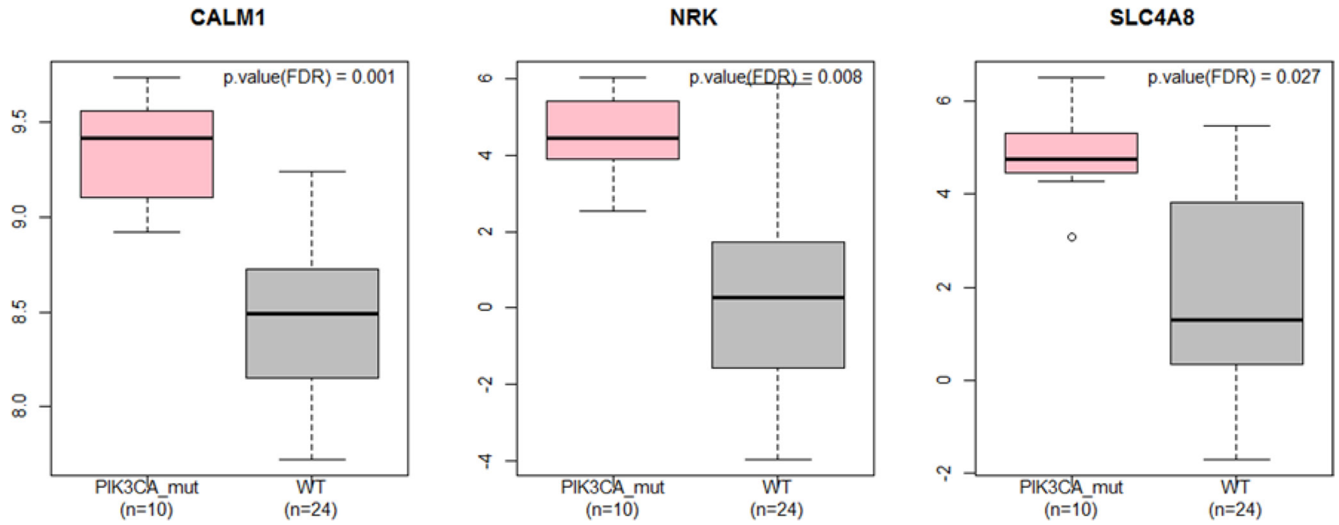

Supplementary Figure 4: The level of gene expression according to *PIK3CA* mutation.

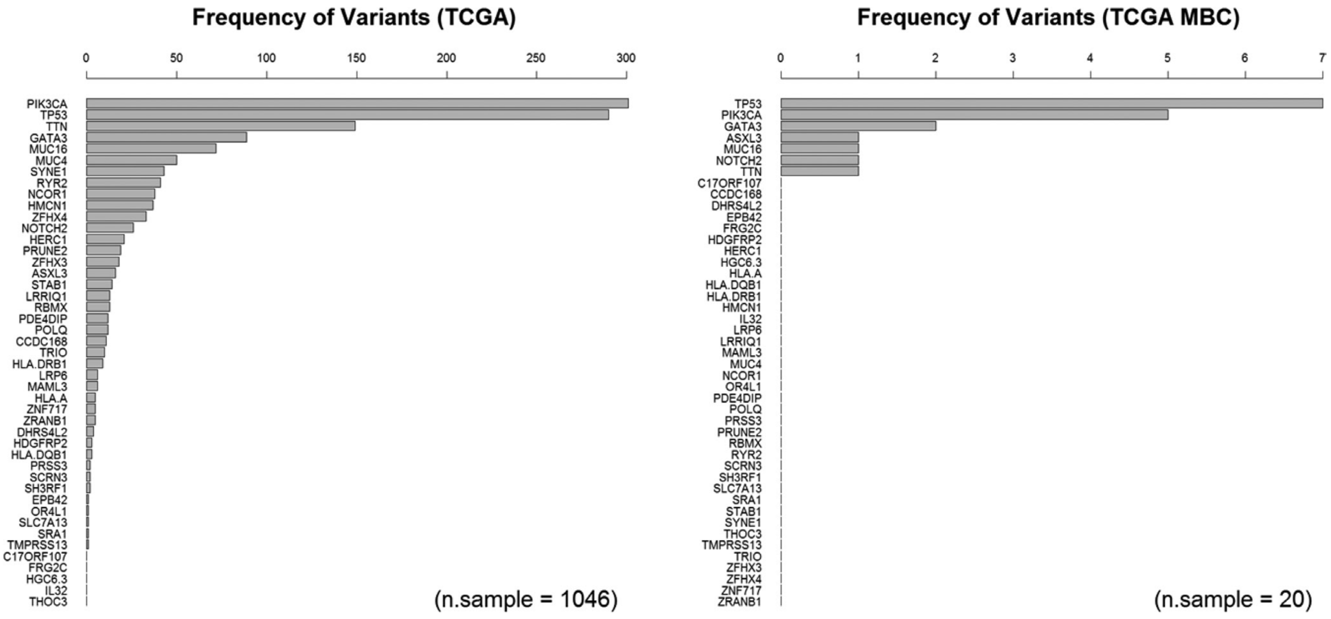

Supplementary Figure 5: (**A**) Somatic mutation profile in the TCGA cohort (**B**) in the TCGA cohort (metastatic cohort).

Supplementary Table 1: Gene expression patterns according to ER status. See Supplementary_ Table_1

Supplementary Table 2: KEGG_DRUG_METABOLISM_OTHER_ENZYMES.
See Supplementary_Table_2

Supplementary Table 3: Baseline characteristics of metastatic breast cancer used for targeted deep seqeuncing (*N* = 29). See Supplementary_Table_3

Supplementary Table 4: List of metastatic breast cancers in the TCGA cohort (*N* = 20)

	ID	er.status	Meta.AJCC	TP53_Mut	PIK3CA_Mut
1	TCGA_5L_AAT1	ER+	M1	-	-
2	TCGA_A2_A0CS	ER+	M1	-	H1047R
3	TCGA_A2_A0SV	ER+	M1	F113_H115del	-
4	TCGA_A2_A0SW	ER+	M1	R273C	-
5	TCGA_A2_A0T2	ER-	M1	Y220C	-
6	TCGA_A8_A07W	ER+	M1	H179R	-
7	TCGA_A8_A08J	ER+	M1	-	-
8	TCGA_A8_A08O	ER+	M1	-	H1047R
9	TCGA_A8_A08T	ER+	M1	-	E545K
10	TCGA_AC_A62V	ER+	M1	-	-
11	TCGA_AN_A0FJ	ER+	M1	V157Afs*24	-
12	TCGA_AO_A0J5	ER+	M1	-	H1047R
13	TCGA_AR_A0TZ	ER+	M1	-	-
14	TCGA_B6_A0I9	indeterminate	M1	C135Y	-
15	TCGA_B6_A0IB	ER+	M1	-	-
16	TCGA_B6_A0X1	ER-	M1	X331_splice	-
17	TCGA_B6_A3ZX	ER-	M1	V216M	-
18	TCGA_BH_A18J	ER+	M1	-	E545K
19	TCGA_BH_A1FH	ER+	M1	-	-
20	TCGA_LL_A73Z	ER+	M1	-	-
21	TCGA_PL_A8LX	<NA>	M1	-	-
22	TCGA_UU_A93S	ER-	M1	-	-
